# Trajectories of systemic immune inflammation index and mortality risk in patients with moderate-to-severe traumatic brain injury: a retrospective cohort study

**DOI:** 10.3389/fneur.2024.1439318

**Published:** 2025-02-12

**Authors:** Zhiyong Tang, Canlin Liao, Zerui Zhuang, Zhennan Xu, Mingfa Liu, Haixiong Xu

**Affiliations:** ^1^Department of Neurosurgery, Shantou Central Hospital, Shantou, Guangdong, China; ^2^Department of Neurosurgery, Sun Yat-sen Memorial Hospital, Sun Yat-sen University, Guangzhou, Guangdong, China

**Keywords:** traumatic brain injury (TBI), neuroinflammation, group-based trajectory modeling (GBTM), inflammatory biomarkers, systemic immune inflammation index (SII), all-cause hospital mortality

## Abstract

**Background:**

Some studies have shown a strong link between the central nervous system and peripheral immune system, but the prognostic implications of dynamic peripheral immune-inflammatory responses in patients with traumatic brain injury (TBI) remain unclear. This study aimed to determine the dynamic trajectory patterns of the Systemic Immune Inflammation Index (SII) in patients with TBI and assess its association with all-cause hospital mortality.

**Methods:**

This retrospective cohort study utilized a large public database of patients with TBI sourced from the eICU Collaborative Research Database (eICU-CRD). Group-Based Trajectory Modeling (GBTM) was used to analyze daily SII trajectories during the initial 0–7 days of hospitalization. Logistic regression was employed to assess the relationship between different SII trajectory groups and hospital mortality. Receiver Operating Characteristic (ROC) curves were generated based on the logistic regression model.

**Results:**

A total of 312 patients were included in this study, 52 of whom died during hospitalization. Using GBTM, three distinct SII trajectories were identified: Group 1 (low-level, rapid decline; 18.90%), Group 2 (moderate-level, slow decline; 60.20%), and Group 3 (sustained high-level; 20.80%). Compared to patients in Group 1, those in Groups 2 and 3 had a higher risk of all-cause hospital mortality (odds ratio [OR] 4.09; 95% confidence interval [CI] 1.21, 19.75) and (OR 5.84; 95% CI 1.52, 30.67), respectively. ROC analysis revealed an area under the curve (AUC) of 0.838, sensitivity: 75.0%, and specificity: 83.8% for mortality in this cohort.

**Conclusion:**

This study identified three distinct SII trajectories, suggesting that post-TBI SII trajectories are heterogeneous patterns associated with mortality. The sustained high-level SII trajectory may serve as a marker of disease deterioration, highlighting the need for targeted interventions. Describing the evolution of SII through GBTM and its correlation with clinical outcomes can enhance our understanding of the link between neuroinflammation and the peripheral immune system.

## 1 Introduction

TBI is a significant cause of mortality and disability globally (1). According to surveillance conducted by the Centers for Disease Control and Prevention (CDC) in the United States, the annual rates of TBI-related emergency department visits and hospitalizations are 403 per 100,000 people and 85 per 100,000 people, respectively (2). The mortality rate for patients with moderate TBI is estimated to be approximately 10%, whereas for patients with severe TBI, it can be as high as 50% (1, 3, 4). To develop personalized treatment plans and minimize the misuse of medical resources, accurate identification of patients with poor prognosis is essential to guide clinical decision-making (5). In the past few decades, TBI research has made rapid progress in the field of biomarkers. These biomarkers play a key role in elucidating pathophysiological processes by examining concentration changes associated with cell damage. Examples of such biomarkers include neuron specific enolase, S100 calcium binding protein B, neurofilament light, and myelin basic protein (6). Currently, there is a high demand for technology to determine low concentrations of routine TBI biomarkers in body fluids, and not all hospitals have the ability to perform these tests. Therefore, it is still imperative to identify and develop novel biomarkers that are easy to obtain and related to the prognosis of TBI.

TBI can be classified into primary and secondary brain injury (7). Secondary injury mechanisms are pivotal in worsening patients’ conditions, with neuroinflammation being recognized as a primary contributing factor (8). Neuroinflammation after TBI is a complex pathophysiological process that involves both resident and peripheral immune cells. Primary traumatic brain injury initiates damage-associated molecular patterns (DAMPs), promoting the production of cytokines and chemokines, and further accelerating the infiltration of peripheral immune cells (9). Following TBI, disruption of the blood–brain barrier (BBB) at the injury site allows direct exchange between the bloodstream and brain. This exchange facilitates the accumulation of platelets and leukocytes in the injured brain tissue, triggering inflammation and immune responses that lead to secondary brain injury (10). Changes in the blood–brain barrier enable neutrophils to migrate to the injury site within the first hour of brain injury (11). Another study indicated that platelet activation correlates with early pro-inflammatory responses following TBI (11, 12). Patients with TBI often show significantly higher absolute numbers and proportions of circulating neutrophils than healthy individuals, with neutrophil counts doubling within 3–4.5 h post-injury (13). Recent studies have explored the predictive ability of systemic inflammatory biomarkers such as the platelet-to-lymphocyte ratio (PLR) and neutrophil-to-lymphocyte ratio (NLR), among others, for prognosis following TBI. These biomarkers have demonstrated promising predictive capabilities (14, 15). Increasing evidence suggests a strong connection and interaction between the central nervous system and the peripheral immune system (16). So, after traumatic brain injury, systemic inflammatory biomarkers may reflect the severity of neuroinflammation and predict the prognosis of TBI patients. However, the previous biomarkers of inflammation were calculated based on two types of immune cells, but this does not fully indicate the role of inflammation in traumatic brain injury. SII, as a ratio calculated based on platelets, neutrophils, and lymphocytes, includes three different types of peripheral blood inflammatory cells compared to other common inflammatory parameters such as NLR, lymphocyte to monocyte ratio (LMR), and PLR. This parameter can more accurately and comprehensively reflect the balance between inflammation and immune response in patients (17).

Recent research has shown that the SII has superior predictive performance compared to NLR, PLR, and LMR in the context of TBI (18, 19). Vascular dysfunction and BBB breakdown after TBI are at the core of thrombosis and immunopathological mechanism development. Platelets play a crucial role in BBB dysfunction, as their activation and aggregation are necessary steps in the process of microthrombus formation, which is closely related to microvascular occlusion and neuronal death (20). Considering the crucial role of platelet activation and aggregation after TBI, as well as their interaction with neutrophils, the SII index can more accurately and comprehensively evaluate the pathological and physiological status after TBI. So that is why SII was chosen as the main focus, and SII may be superior to other inflammatory markers. In these studies, researchers primarily focused on the relationship between a single time point SII and the outcomes of patients with TBI, overlooking the dynamic assessment of systemic inflammation. There has been less emphasis on evaluating the relationship between neuroinflammation over time and prognosis, as well as the diversity and heterogeneity of secondary injuries (21). The SII at a certain time point may not reflect the pathological and physiological changes after early TBI well. GBTM is a statistical method used to identify clusters of individuals with similar longitudinal patterns of biomarker progression over time (22). By estimating changes in repeated measurements over time, GBTM can identify distinct clusters of individuals exhibiting similar longitudinal change patterns. This modeling approach involves finite mixture models of unobserved subpopulations, with assumptions about trajectory shapes, allowing for the testing of different trajectory groups through maximum likelihood estimation (23).

The trajectory of the SII has not yet been studied in TBI, and dynamic SII trajectories could reflect the pathophysiological changes in disease progression. Exploring SII trajectory patterns can help uncover the heterogeneity of the inflammatory response and dynamic changes of SII during the early stages of TBI, identify individuals at high risk of mortality, and provide new insights into treatment strategies. This study aimed to utilize GBTM to identify SII trajectories in patients with moderate-to-severe TBI and further evaluate the association between SII trajectories and the risk of in-hospital all-cause mortality.

## 2 Methods

### 2.1 Data source and ethics compliance

This study utilized a large-scale publicly available database, specifically the eICU-CRD version 2.0 (24). The eICU-CRD is a large multicenter database comprising data covering over 200,000 ICU admissions from 2014 to 2015 in the United States. Patients were admitted to one of 335 units at 208 hospitals located throughout the US. The database is deidentified, and includes vital sign measurements, care plan documentation, severity of illness measures, diagnosis information, laboratory measurements, patient history, treatment information, and more. Access to this database was granted to one (TZY) of the authors upon completion of the collaborative institutional training initiative exam (Certification ID: 50723731), who was responsible for data extraction. This was an observational study. Data were extracted from the eICU-CRD version 2.0. Ethical approval and informed consent were waived because all information was anonymized and the re-identification risk was certified as meeting safe harbor standards by an independent privacy expert (Privacert, Cambridge, MA; Health Insurance Portability and Accountability Act Certification No. 1031219–2).

### 2.2 Population selection criteria

The inclusion criteria were patients with TBI as their primary or major diagnosis. For patients with multiple admissions, data from their initial hospitalization were used. Patients with a Glasgow Coma Scale (GCS) score > 12, aged <16 or > 89 years, diagnosed with infectious diseases or hematologic malignancies, undergoing chemotherapy, using medications severely affecting blood cell counts, or those with missing survival data were excluded. Additionally, patients with less than 3 days of SII data were excluded from the GBTM analysis.

### 2.3 Clinical data collection

We used PostgreSQL software (version 15) and Navicate Premium software (version 15.0.29) with structured query language (SQL) to extract data from the eICU-CRD for the years 2014 to 2015. All the codes for computing demographic features, laboratory tests, comorbidities, and severity scores were obtained from the GitHub website.^1^ This study gathered demographic and clinical data as well as comorbidities, and then calculated the Charlson Comorbidity Index (CCI). Demographic information included age, sex, race, and body mass index (BMI). The study also recorded specific injury types (such as skull fractures, epidural hematoma, subarachnoid hemorrhage, subdural hematoma, contusion or laceration, and intracerebral hemorrhage) alongside multiple scoring systems [including GCS, Sequential Organ Failure Assessment (SOFA), and Acute Physiology Score III (APS III)]. Data also encompassed medication treatments, laboratory indicators, vital signs, length of hospital stay, neurosurgical interventions, and outcome indicators (hospital mortality). Based on previous studies (25, 26), some variables were repeatedly measured within the initial 24 h of admission, and the value that best reflected the severity of the disease was used. The primary outcome of this study was all-cause hospital mortality, with the exposure factor being the longitudinal trajectory of SII. Variables with a missing proportion exceeding 10% were excluded from our analysis. Missing values are processed using Multiple Imputation by Chained Equations (MICE) algorithm (Supplementary Table S1). Complete blood counts for the initial 7 days post-admission were included in our analysis. SII = platelet × neutrophil/lymphocyte.

### 2.4 Statistical analysis

Continuous variables were presented as either medians with interquartile ranges (IQR) or means with standard deviations (SD), while categorical variables were described as counts and percentages. The normality of variables was assessed using the Shapiro–Wilk test. For comparisons involving categorical variables, either the chi-square test or Fisher’s exact test was employed. At the same time, analysis of variance was utilized for continuous variables following a normal distribution to compare different SII trajectory patterns. The Kruskal–Wallis test was used for continuous variables with non-normal distributions to compare the different trajectory patterns.

GBTM is a semi-parametric finite mixture model capable of identifying groups of individuals with similar biomarker progression (22). This method assumes that the population consists of a finite number of distinct groups defined by their biomarker trajectories. PROC TRAJ is a SAS program suitable for GBTM (27). It provides the ability to model three different data distributions for the variable of interest: (1) counts; (2) continuous data; and (3) dichotomous data. Since SII is a continuous variable and biomarkers typically exhibit skewed distributions, we log-transformed SII. We then used GBTM with a censored normal distribution (CNORM) to identify subgroups of participants with different SII trajectories within the first 7 days post-trauma. One key decision when identifying trajectory groups in a population is determining the optimal number of groups to best fit the data. One must also decide on the highest polynomial order that best describes the trajectory of the biomarker over time for each group. The polynomial order relates to the shape of the trajectory. Models with different numbers of groups and shapes must be compared to identify the model that best fits the biomarker data. Several model fit indices can help determine the best model, but one commonly used measure is the Bayesian Information Criterion (BIC). When comparing two possible trajectory models (e.g., with different numbers of groups and/or trajectory shapes [polynomial orders]), the model with the higher BIC value is selected. If two models fit the data similarly, but one is more complex (i.e., has more groups or higher-order polynomials) than the other, the simpler model should be chosen (28). Akaike Information Criterion (AIC) and log-likelihood (LL) also assist in selecting the optimal model, and like BIC, the model with the highest AIC and LL values is preferred. Average posterior probability (AvePP): If individuals are unambiguously assigned to different groups, the AvePP for each group would be 1. Therefore, the closer the AvePP is to 1, the better the model fit. It is generally recommended that the AvePP for all groups be greater than 0.7. Odds of Correct Classification (OCC): For a model that fits the data well, the OCC values should be significantly greater than 1. It is generally recommended that the OCC for all groups be 5 or higher (29). We employed the standard approach provided by the BIC to determine the optimal number of trajectory groups. We iteratively refined the model by eliminating non-significant polynomial terms, reducing polynomial levels to achieve a simplified final model, where the significance level for each trajectory group was set at *α* ≤ 0.05 (23, 30). Models ranging from two to six trajectory groups were fitted using linear, quadratic, and cubic polynomial terms. Typically, we started with a model consisting of two groups with cubic polynomials, then progressively eliminated non-significant polynomial terms, reducing the polynomial order until the highest-order polynomial for each group was significant at a confidence level of *α* ≤ 0.05. We then increased the number of groups and repeated this process for models with three to six groups. The selection of the best trajectory was based on various parameters, including the AvePP, Occ, BIC, AIC, and LL. We selected models with higher BIC, AIC, and LL values and AvePP ≥0.7, Occ >5, while also favoring simpler models (31, 32) (Supplementary Figure S1, Table S2).

After identifying the optimal SII trajectory groups, we used logistic regression models to examine the association between SII trajectory and all-cause hospital mortality. Initially, a crude logistic regression model was established with the first group as the reference. Subsequently, we constructed a multivariable logistic regression model. In the process of constructing a multivariable logistic regression model, we first performed univariable logistic regression analysis on variables including demographics, Charlson Comorbidity Index, injury type, medication, laboratory indicators, vital signs, length of hospital stay, neurosurgical interventions, and scoring systems. Variables with a significance level of *p* < 0.05 from the univariable analysis were then included in the multivariable logistic regression model. To reduce model complexity, avoid overfitting, and ensure that only variables with a significant effect on the outcome variable were included in the model, stepwise regression was used to further select variables. This helped identify the optimal model and ensure minimal multicollinearity. Variables with *p* < 0.05 in the stepwise regression were incorporated into the final multivariable logistic regression model for adjustment of confounding factors. Additionally, other commonly recognized risk factors, such as age, GCS, and surgical treatment, were also included in the final multivariable logistic regression model. If variance inflation factor (VIF) ≥ 10, it is considered that there is collinearity between variables. In this analysis, all VIF values were below 10, suggesting that no significant multicollinearity was detected. Consequently, no variables were excluded from the model. ROC curve analysis was conducted based on multivariable logistic regression model. Sensitivity analysis was performed based on the severity of traumatic brain injury, excluding patients with moderate TBI and reconstructing the sensitivity analysis cohort. An additional GBTM model was developed to explore the association between subgroups in this model and all-cause hospital mortality.

Two-sided *p*-value <0.05 was considered statistical significance. We did all analyses with SAS version 9.4 software and R version 4.2.2 software.

## 3 Results

### 3.1 Study cohort

**Figure 1:**
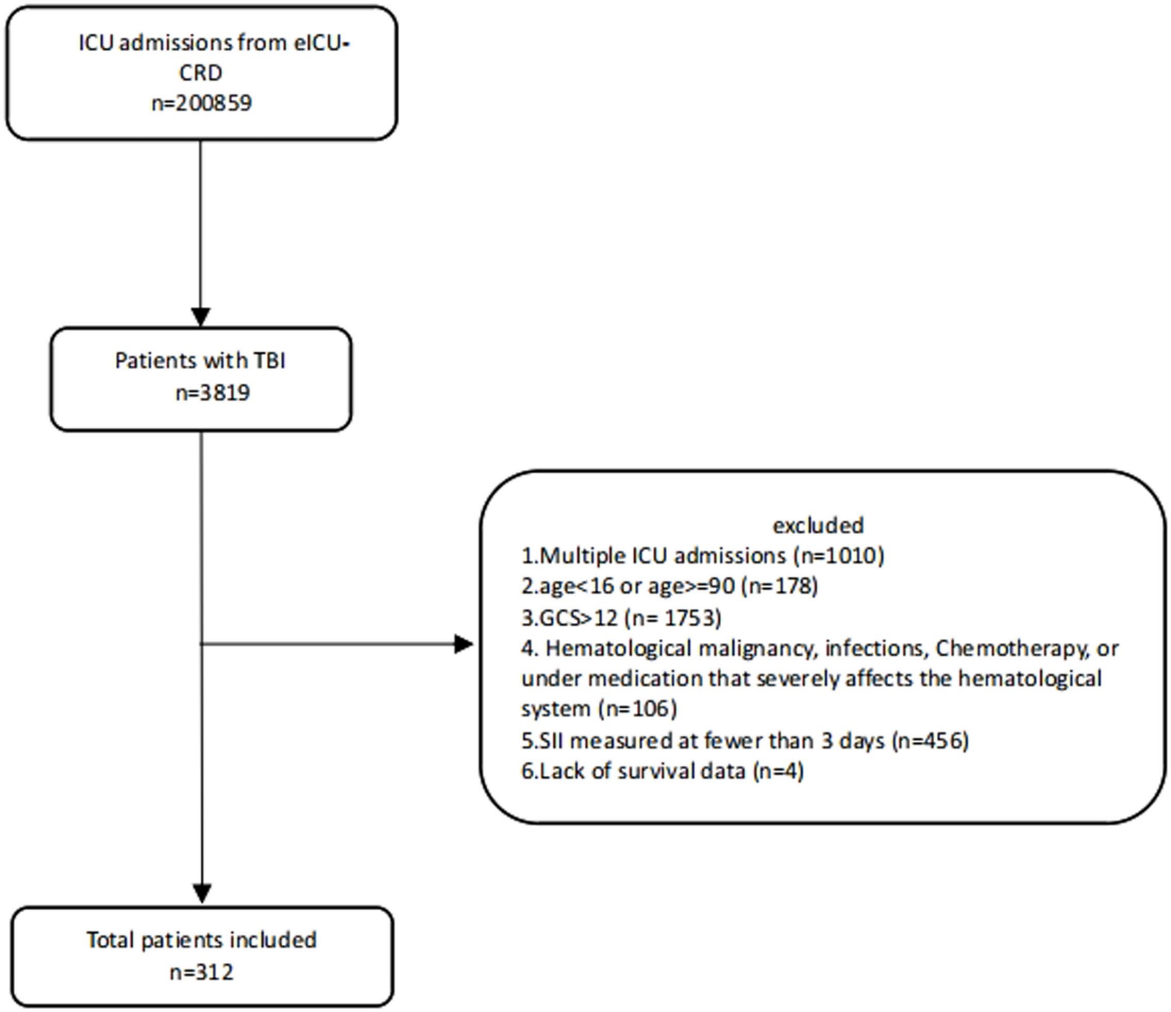
Flowchart of eligible participants.

Initially, a total of 200,859 ICU admission records were retrieved from the eICU-CRD database. After applying exclusion criteria, 312 patients were included in the study cohort, with a total of 1,757 SII measurements conducted within 7 days post-trauma (Figure 1). Among them, 52 cases (16.7%) died during hospitalization. The median age of the patients was 54 years (IQR 32.80–70.00), with males comprising 66.7% of the cohort. The median Min GCS score was 6 (IQR 3.00–8.00), and the primary TBI type was subdural hematoma (40.01%). Compared to survivors, non-survivors had higher Max creatinine and Max glucose levels [1.09 mg/dL (IQR 0.84, 1.43) vs. 0.90 mg/dL (IQR 0.77, 1.10); 188 mmol/L (IQR 157, 244) vs. 156 mmol/L (IQR 133, 195)], elevated Max white blood cell count (WBC) [17.80 (IQR 11.8, 23.8) vs. 15.00 (IQR 10.6, 18.2)], lower Min systolic blood pressure (SBP) and Min diastolic blood pressure (DBP) [(86.2 mmHg, SD ± 23.6) vs. (99.7 mmHg, SD ± 20.8); (45.6 mmHg, SD ± 15.5) vs. (51.5 mmHg, SD ± 12.6)], and reduced Min GCS (3.00 [IQR 3.00, 6.00] vs. 6.00 [IQR 3.00, 8.00]). Additionally, a higher proportion of non-survivors received mannitol (23.1% vs. 6.15%) and underwent ventriculostomy (23.1% vs. 10.4%; Supplementary Table S3).

### 3.2 Characterization of SII trajectories

**Figure 2:**
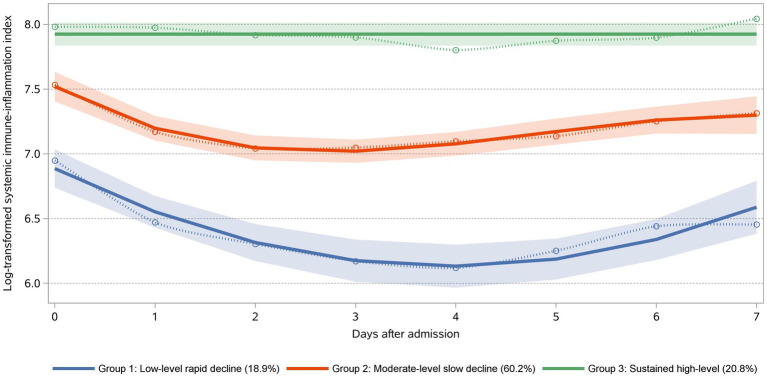
SII trajectories within the first 7 days post-admission following trauma. Trajectories were defined using group-based trajectory modeling. Day 0 was defined as the day of admission. Group 1: “Low-Level Rapid Decline” (18.90%) exhibited the lowest mortality rate. Group 2: “Moderate-Level Slow Decline” (60.20%) demonstrated a moderate risk of mortality. Group 3: “Sustained High-Level” (20.80%) was associated with the highest mortality rate.

Our model identified three distinct longitudinal trajectories of daily maximum SII (Figure 2). The AvePP for each group exceeded 0.85, and the proportion of the population was greater than 5%. These three trajectory groups reflected different patterns of daily maximum SII values post-trauma. Group 1 (*n* = 59, 18.90%) represents the low-level rapid decline group, initially characterized by the low level, sharply decreasing to its minimum around the 4th day, followed by a slight rebound. Group 2 (*n* = 188, 60.20%) represented the moderate-level slow decline group, being the largest cohort, exhibiting an initial gradual decline, followed by a rebound, and ultimately stabilizing. Group 3 (*n* = 65, 20.80%) represents the sustained high-level group, displaying the highest Log SII values and maintaining consistently elevated levels without a clear declining pattern.

### 3.3 Clinical characteristics

Table 1 presents the differences in demographic and clinical characteristics among different SII trajectory groups. Group 2 has the highest proportion of males (70.70%), which is higher than in the other two groups. There were no differences in age, race, and BMI among the three groups, and the maximum WBC and platelet counts were higher in Group 2 and Group 3 than in Group 1 (Max WBC: 15.40 [IQR 10.70, 19.00] and 15.40 [IQR 11.50, 20.60]; Max platelets: 226.00 [IQR 176.00, 282.00] and 260.00 [IQR 241.00, 320.00] for Groups 2 and 3, respectively). There were differences in the types of TBI among the three trajectory groups, with subdural hematoma being predominant in Group 3 (50.80%). Additionally, the proportion of patients undergoing surgical treatment and receiving mannitol was highest in Group 3 (24.60 and 13.80%, respectively), but these differences were not statistically significant. Differences were observed in the maximum, minimum, and mean values of SII among the three groups, with Group 3 exhibiting higher than the other two groups.

**Table 1:** Characteristics among trajectory groups.

	ALL (*N* = 312)	Group1 (*N* = 59)	Group2 (*N* = 188)	Group3 (*N* = 65)	*p*
Demographics					
Age (years, median [IQR])	54.0 [32.8;70.0]	50.0 [35.0;65.0]	53.5 [30.0;66.0]	56.0 [35.0;77.0]	0.216
Sex, n (%)					**0.044**
Female	104 (33.3%)	19 (32.2%)	55 (29.3%)	30 (46.2%)	
Male	208 (66.7%)	40 (67.8%)	133 (70.7%)	35 (53.8%)	
Ethnicity, n (%)					0.566
African American	21 (6.73%)	4 (6.78%)	13 (6.91%)	4 (6.15%)	
Asian	5 (1.60%)	0 (0.00%)	3 (1.60%)	2 (3.08%)	
Caucasian	241 (77.2%)	48 (81.4%)	145 (77.1%)	48 (73.8%)	
Hispanic	21 (6.73%)	2 (3.39%)	11 (5.85%)	8 (12.3%)	
Native American	2 (0.64%)	1 (1.69%)	1 (0.53%)	0 (0.00%)	
Other/Unknown	22 (7.05%)	4 (6.78%)	15 (7.98%)	3 (4.62%)	
BMI(kg/m^2^,median [IQR])	26.4 [22.9;29.9]	27.4 [22.5;29.8]	26.4 [23.4;30.3]	25.7 [22.1;29.8]	0.227
CCI, median (IQR)	2.00 [0.00;4.00]	2.00 [0.00;4.00]	2.00 [0.00;4.00]	3.00 [0.00;4.00]	0.221
Laboratory variates, median (IQR)					
Min creatinine (mg/dL)	0.78 [0.63;0.95]	0.78 [0.64;0.90]	0.78 [0.62;0.94]	0.83 [0.64;0.97]	0.886
Max creatinine (mg/dL)	0.92 [0.78;1.14]	0.90 [0.80;1.08]	0.90 [0.78;1.14]	0.94 [0.77;1.17]	0.728
Min glucose (mmol/L)	108 [93.0;127]	105 [92.0;122]	110 [94.8;127]	109 [94.0;129]	0.348
Max glucose (mmol/L)	160 [135;202]	150 [128;186]	161 [136;208]	162 [140;207]	0.193
Min Hb (g/dL)	11.4 [9.30;12.7]	11.7 [9.20;13.5]	11.6 [9.57;12.8]	10.5 [9.10;12.2]	0.101
Max Hb (g/dL)	13.3 [11.8;14.6]	13.5 [11.6;14.9]	13.4 [12.1;14.6]	12.6 [10.8;14.2]	0.084
Min INR (ratio)	1.10 [1.00;1.20]	1.10 [1.05;1.20]	1.10 [1.00;1.20]	1.10 [1.01;1.20]	0.321
Max INR (ratio)	1.17 [1.09;1.30]	1.14 [1.10;1.37]	1.16 [1.05;1.30]	1.20 [1.10;1.36]	0.767
Min platelet (10^9^/L)	174 [134;223]	158 [110;182]	169 [132;220]	204 [167;268]	**<0.001**
Max platelet (10^9^/L)	223 [178;280]	194 [169;222]	226 [176;282]	260 [214;320]	**<0.001**
Min potassium (mmol/L)	3.50 [3.20;3.80]	3.60 [3.30;3.80]	3.50 [3.20;3.80]	3.50 [3.20;3.80]	0.43
Max potassium (mmol/L)	4.10 [3.90;4.60]	4.10 [3.90;4.45]	4.10 [3.80;4.60]	4.20 [3.90;4.50]	0.558
Min sodium (mmol/L)	138 [136;140]	139 [136;140]	138 [136;140]	138 [135;141]	0.727
Max sodium (mmol/L)	142 [139;145]	141 [139;144]	142 [139;145]	142 [140;145]	0.504
Min WBC (10^9^/L)	10.5 [8.13;13.7]	9.50 [8.00;12.1]	10.3 [7.97;13.4]	12.5 [9.20;15.6]	**0.005**
Max WBC (10^9^/L)	15.2 [10.8;18.9]	12.0 [9.90;16.6]	15.4 [10.7;19.0]	15.4 [11.5;20.6]	**0.035**
Min NEU (10^9^/L)	8.02 [5.65;10.9]	6.93 [5.24;9.62]	7.89 [5.64;10.8]	9.63 [7.36;12.6]	**0.002**
Max NEU (10^9^/L)	11.4 [8.13;15.3]	9.83 [7.41;13.1]	11.8 [8.12;15.4]	12.4 [9.48;16.8]	**0.015**
Min LYM (10^9^/L)	1.02 [0.68;1.46]	1.30 [1.00;1.88]	0.97 [0.66;1.46]	0.86 [0.63;1.23]	**<0.001**
Max LYM (10^9^/L)	1.73 [1.13;2.97]	1.95 [1.37;2.95]	1.69 [1.12;3.16]	1.51 [0.94;2.72]	0.113
Min MON (10^9^/L)	0.72 [0.53;0.97]	0.74 [0.53;1.00]	0.72 [0.53;0.95]	0.76 [0.53;1.01]	0.756
Max MON (10^9^/L)	1.00 [0.74;1.40]	1.00 [0.80;1.26]	0.99 [0.74;1.42]	1.02 [0.68;1.44]	0.933
Vital Signs, median (IQR)					
Min heart rate (times/min)	67.0 [58.0;77.0]	66.0 [58.0;74.5]	67.0 [59.0;78.0]	67.0 [55.0;73.0]	0.463
Max heart rate (times/min)	110 [96.8;128]	111 [96.5;127]	110 [96.8;126]	117 [98.0;137]	0.33
Min SBP (mmHg), means (SD)	97.7 (21.7)	95.9 (15.9)	98.6 (23.2)	96.7 (22.0)	0.641
Max SBP (mmHg)	161 [147;180]	157 [136;178]	160 [149;179]	168 [146;182]	0.142
Min DBP (mmHg), means (SD)	50.5 (13.2)	50.7 (10.2)	51.4 (13.6)	47.8 (14.3)	0.157
Max DBP (mmHg)	97.5 [84.0;113]	97.0 [84.5;108]	98.0 [85.0;113]	95.0 [81.0;112]	0.775
Min temperature (°C)	36.3 [35.7;36.7]	36.2 [35.8;36.7]	36.3 [35.7;36.7]	36.4 [35.6;36.6]	0.869
Max temperature (°C), means (SD)	38.0 (0.72)	37.9 (0.76)	38.0 (0.69)	38.1 (0.76)	0.381
Type of TBI, n (%)					
Skull fracture					0.858
No	299 (95.8%)	56 (94.9%)	180 (95.7%)	63 (96.9%)	
Yes	13 (4.17%)	3 (5.08%)	8 (4.26%)	2 (3.08%)	
EDH					0.315
No	295 (94.6%)	57 (96.6%)	179 (95.2%)	59 (90.8%)	
Yes	17 (5.45%)	2 (3.39%)	9 (4.79%)	6 (9.23%)	
tSAH					0.472
No	215 (68.9%)	44 (74.6%)	125 (66.5%)	46 (70.8%)	
Yes	97 (31.1%)	15 (25.4%)	63 (33.5%)	19 (29.2%)	
SDH					**0.027**
No	187 (59.9%)	43 (72.9%)	112 (59.6%)	32 (49.2%)	
Yes	125 (40.1%)	16 (27.1%)	76 (40.4%)	33 (50.8%)	
tICH					0.367
No	292 (93.6%)	57 (96.6%)	173 (92.0%)	62 (95.4%)	
Yes	20 (6.41%)	2 (3.39%)	15 (7.98%)	3 (4.62%)	
Contusion or laceration					0.465
No	230 (73.7%)	45 (76.3%)	134 (71.3%)	51 (78.5%)	
Yes	82 (26.3%)	14 (23.7%)	54 (28.7%)	14 (21.5%)	
Scoring Systems, median (IQR)					
Min GCS	6.00 [3.00;8.00]	6.00 [3.00;8.00]	4.00 [3.00;8.00]	6.00 [3.00;7.00]	0.868
SOFA	4.00 [3.00;6.00]	4.00 [3.00;7.50]	4.00 [3.00;6.00]	4.00 [3.00;6.00]	0.929
APSIII	53.0 [37.0;74.0]	47.0 [34.5;74.5]	54.0 [35.0;73.2]	54.0 [40.0;74.0]	0.404
Clinical Treatments, n (%)					
Ventriculostomy					0.139
No	273 (87.5%)	56 (94.9%)	160 (85.1%)	57 (87.7%)	
Yes	39 (12.5%)	3 (5.08%)	28 (14.9%)	8 (12.3%)	
Craniotomy					0.159
No	287 (92.0%)	55 (93.2%)	176 (93.6%)	56 (86.2%)	
Yes	25 (8.01%)	4 (6.78%)	12 (6.38%)	9 (13.8%)	
CSF drainage					0.163
No	286 (91.7%)	57 (96.6%)	168 (89.4%)	61 (93.8%)	
Yes	26 (8.33%)	2 (3.39%)	20 (10.6%)	4 (6.15%)	
Craniectomy					0.838
No	308 (98.7%)	59 (100%)	185 (98.4%)	64 (98.5%)	
Yes	4 (1.28%)	0 (0.00%)	3 (1.60%)	1 (1.54%)	
Surgery					0.168
No	248 (79.5%)	52 (88.1%)	147 (78.2%)	49 (75.4%)	
Yes	64 (20.5%)	7 (11.9%)	41 (21.8%)	16 (24.6%)	
Hypertonic saline					0.155
No	268 (85.9%)	55 (93.2%)	160 (85.1%)	53 (81.5%)	
Yes	44 (14.1%)	4 (6.78%)	28 (14.9%)	12 (18.5%)	
Mannitol					0.055
No	284 (91.0%)	58 (98.3%)	170 (90.4%)	56 (86.2%)	
Yes	28 (8.97%)	1 (1.69%)	18 (9.57%)	9 (13.8%)	
Hospital Outcomes					
LOS hospital hours, median (IQR)	249 [148;420]	229 [148;399]	250 [144;416]	290 [176;498]	0.345
In-hospital death, n (%)					**0.013**
No	260 (83.3%)	56 (94.9%)	155 (82.4%)	49 (75.4%)	
Yes	52 (16.7%)	3 (5.08%)	33 (17.6%)	16 (24.6%)	
SII Characteristics, median (IQR)					
Avg SII	1,557 [1,014;2,236]	768 [622;856]	1,524 [1,210;1900]	3,089 [2,615;3,675]	**<0.001**
Min SII	782 [546;1,123]	365 [280;477]	788 [642;1,038]	1701 [1,286;2,112]	**<0.001**
Max SII	2,622 [1,666;4,216]	1,367 [915;1,690]	2,531 [1792;3,596]	5,287 [4,002;7,452]	**<0.001**

BMI, body mass index; CCI, Charlson comorbidity index; Hb, hemoglobin; INR, International Normalized Ratio; WBC, white blood cells; NEU, neutrophile; LYM, lymphocyte; MON, monocyte; SBP, systolic blood pressure; DBP, diastolic blood pressure; TBI, traumatic brain injury; EDH, epidural hematoma; SDH, subdural hematoma; tSAH, traumatic subarachnoid hemorrhage; tICH, traumatic intracerebral hemorrhage; GCS, Glasgow Coma Score; SOFA, sequential organ failure assessment; APSIII, acute physiology score III; CSF, cerebrospinal fluid; LOS, length of stay; SII, systemic immune inflammation index. Boldface type indicates *p* < 0.05.

### 3.4 Association of SII trajectories and all-cause hospital mortality

**Figure 3:**
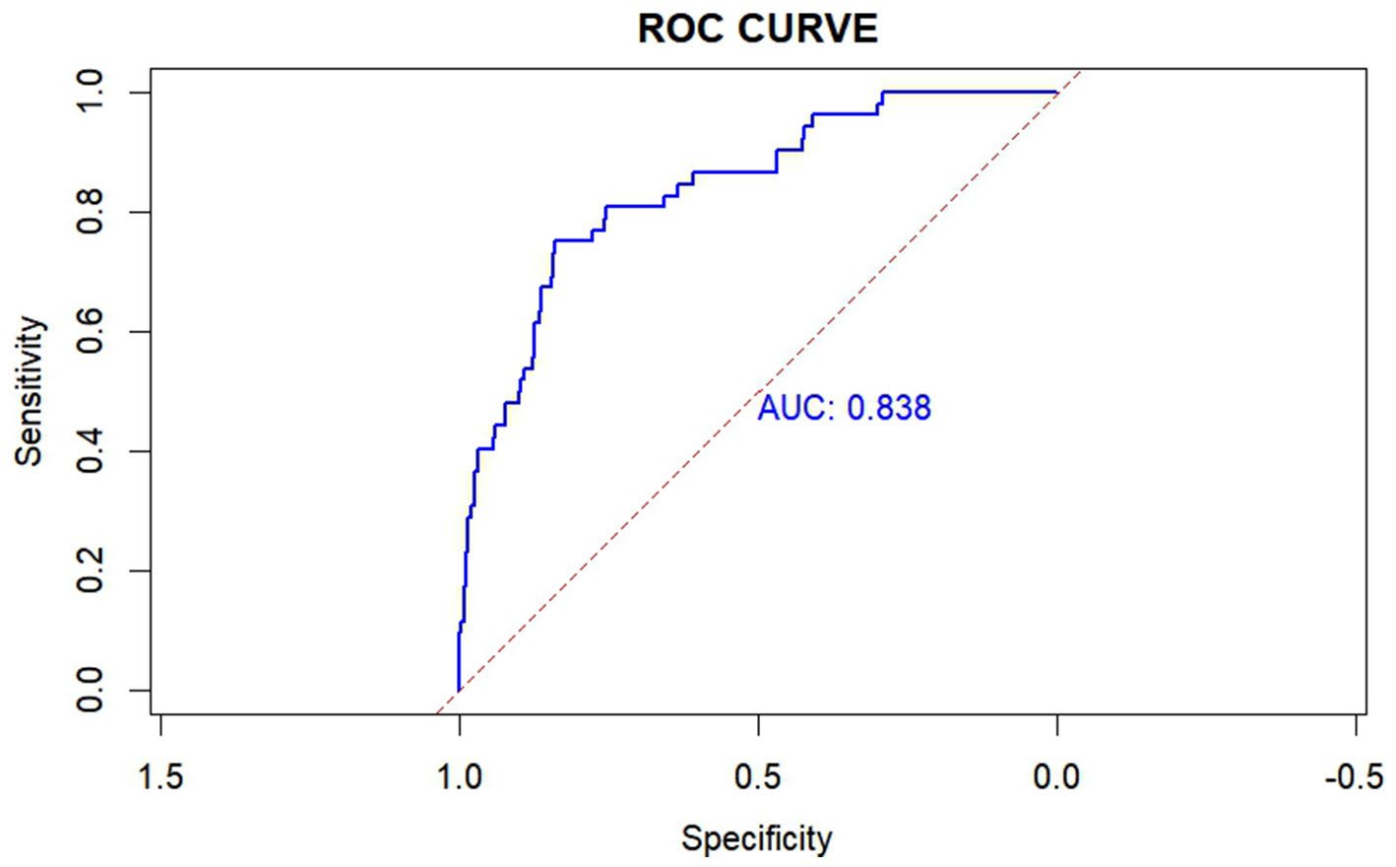
Receiver operating characteristic (ROC) curve of the SII trajectory model. This ROC curve achieved an area under the curve (AUC) of 0.838, with a sensitivity of 75.0% and a specificity of 83.8%. The SII trajectory model was adjusted using APSIII, Max creatinine, Min SBP, Mannitol, Age, Min GCS, Surgery. AUC: the area under the receiver-operating-characteristics curve.

There was a significant difference in all-cause hospital mortality rates among the different trajectory groups (*p* = 0.013), with Group 3 showing a higher mortality rate than the other two groups. The mortality rates for Groups 1, 2, and 3 were 5.08, 17.55, and 24.62%, respectively. In the unadjusted model, compared to Group 1, belonging to Groups 2 and 3 was associated with increased mortality (OR 3.97, 95% CI 1.36–16.97; OR 6.01, 95% CI 1.89–27.33). After adjusting for potential confounders (APSIII, Max creatinine, Min SBP, Mannitol, Age, Min GCS, Surgery), the multivariable logistic regression model yielded similar results, showing an increased risk of mortality in Groups 2 and 3 (OR 4.09, 95% CI 1.21–19.75; OR 5.84, 95% CI 1.52–30.67; Table 2). Subsequently, we calculated the model’s AUC to be 0.838, sensitivity: 75.0%, and specificity: 83.8% (Figure 3).

**Table 2:** Association between SII trajectories and all-cause hospital mortality.

Trajectories	Univariate analysis		Multivariate analysis	
	OR (95% CI)	*p-*value	OR (95% CI)	*p*-value
Group 1	Reference		Reference	
Group 2	3.97(1.36,16.97)	**0.027**	4.09 (1.21,19.75)	**0.042**
Group 3	6.01(1.89,27.33)	**0.006**	5.84 (1.52,30.67)	**0.018**

Adjusted for APSIII, Max creatinine, Mannitol, Min SBP, Age, Min GCS, Surgery. OR, odds ratio; CI, confidence interval. Boldface type indicates *p* < 0.05.

### 3.5 Sensitivity analysis

After excluding patients with moderate TBI, we remodeled and identified a Log SII trajectory plot that closely mirrored the number and evolution patterns of the original cohort. We conducted a rough comparison of the three Log SII trajectory groups and used a logistic regression model to explore the relationship between these trajectory groups and all-cause hospital mortality. The results were consistent with those of the original cohort. Using Group 1 as a reference, the risk of death increased in Group 3 (OR 3.10, 95% CI 0.56, 29.33). Refer to the Supplementary Materials (Supplementary Figure S2, Table S4) for further details.

## 4 Discussion

Post-traumatic brain injury secondary damage has long been a focal point, particularly with neuroinflammation playing a pivotal role. In this study, we employed GBTM to examine dynamic SII patterns following TBI. We identified three distinct trajectory groups based on daily maximum SII levels. Compared to the “low-level rapid decline” and “moderate-level slow decline” groups, the group exhibiting a “sustained high-level” trajectory pattern showed a significantly increased risk of in-hospital mortality. This relationship was confirmed in a multivariable logistic regression model.

Neuroinflammation in the secondary injury following TBI is recognized as one of the important factors influencing disease progression, becoming a major focus of research in recent years (16). In response to brain injury, inflammatory cells from both the central nervous system and periphery rapidly react and may aid in the healing process (33). This inflammatory response is triggered by damaged neuronal tissue, leading to the production of pro-inflammatory cytokines and angiogenic factors (34). Increased levels of cytokines and chemokines, such as tumor necrosis factor (TNF), interleukin (IL)-1β, and IL-6, may serve as signals of the inflammatory response. Their release and production leads to local immune reactions in the brain tissue and systemic immune responses. These cytokines and chemokines result in the recruitment of neutrophils, activation of monocytes, and polarization of microglia and astrocytes (13). In this process, the production of proteases, metalloproteinases, TNF, and reactive oxygen species (ROS) further breaks the BBB, allowing neutrophils to enter the central nervous system (35). Platelets play a critical role in blood–brain barrier dysfunction through their activation and aggregation, which are essential for microthrombus formation associated with microvascular occlusion and neuronal death. Platelets and leukocytes can form microvascular thrombi around the injury site and adjacent brain tissue by forming activated vascular hemophilia factor polymers (36). Therefore, the infiltration of inflammatory cells following TBI plays an indispensable role in traumatic brain injury.

Previous studies have highlighted numerous biomarkers for TBI diagnosis and prognosis, including protein biomarkers for astroglial cell injury (GFAP, S100B), neuronal cell body injury (UCH-L1, NSE), markers of post-injury neurodegeneration (total Tau and phosphorylated Tau), neuronal cell death (alpha-II spectrin breakdown products), axonal injury (NF protein), white matter injury (MBP), and markers of post-injury autoimmune response (brain-targeted autoantibodies). However, these biomarkers may not be universally accessible across all hospitals (37). Therefore, researchers have been striving to identify simple, readily available, and relatively reliable biomarkers. The SII may be associated with adverse outcomes in neurological diseases, such as the severity of pneumonia in patients with intracerebral hemorrhage, the prognosis of patients with acute ischemic stroke, and delayed cerebral vasospasm following aneurysmal subarachnoid hemorrhage (38–40). Studies focusing on patients with TBI have also shown that elevated SII levels upon admission are correlated with poor outcomes. Notably, SII demonstrates superior predictive performance compared to other ratios like PLR, NLR, and LMR (18, 19).

In this study, we focused on patients with a GCS score of ≤12 upon admission, aiming to explore the relationship between inflammatory biomarker trajectories and mortality of patients with moderate-to-severe TBI. Compared to the static assessments of the SII, our research underscores the importance of SII trajectory patterns in patients with TBI. Previous studies have demonstrated that elevated SII levels correlate with poor outcomes upon admission in patients with TBI (18, 19). However, these studies solely examined the association between SII at a single time point and prognosis. Single-time-point assessments of SII may introduce bias owing to varying disease time frames and offer limited insights into disease progression post-TBI. A one-time SII measurement may not fully capture the comprehensive pattern of inflammatory changes during hospitalization. Clinical treatment options for patients with TBI are often limited, as the lack of treatment options stems from the diversity of injury types and an incomplete understanding of secondary brain injury mechanisms (1, 41, 42). Hence, there is an urgent need for methods to explore inflammatory changes following TBI. This exploration can aid in comprehending the pathophysiological mechanisms of inflammation-related secondary injuries and identifying patient heterogeneity post-TBI. By gaining insights into inflammation progression after TBI, clinicians can better comprehend patient conditions, tailor clinical interventions more precisely to individual patients, enhance patient outcomes, and provide a research roadmap for pathophysiological investigation.

Through clinical characteristics and dynamic changes in SII trajectories, we can gain insights into the complex mechanisms of neuroinflammation progression in patients with TBI. For example, patients in Group 3, as opposed to those in Groups 1 and 2, demonstrated higher SII measurements during the first 7 days after admission, with no downward trend. This subgroup exhibited the highest mortality rates, a higher APSIII, and a greater incidence of subdural hematoma, along with increased rates of surgical treatment and mannitol usage. Traumatic acute subdural hematoma (ASDH) is the most severe subtype of TBI due to its high rates of disability and mortality. The lethality of traumatic ASDH primarily arises from its frequent association with primary and/or secondary brain injuries, including contusions, lacerations, edema, or swelling (43). Following traumatic brain injury, the release of molecular patterns associated with primary and secondary injuries triggers an immune response, leading to BBB disruption and vasogenic edema following TBI. These reactions can cause further increases in intracranial pressure (ICP) (44). In our study, Group 3, which exhibited sustained high SII levels, was associated with higher rates of mannitol use, a greater proportion of subdural hematomas, and higher surgical intervention rates. These patients likely had more severe conditions, exhibiting a stronger inflammatory response and cerebral edema, suggesting that they were more prone to severe complications such as increased ICP. The differences in mannitol usage, proportion of subdural hematomas, and surgical interventions indicate that SII trajectories may reflect the severity of the patient’s condition, as well as the need for ICP management and surgical treatment. This aligns with our conclusion that patients with sustained high SII levels had the highest all-cause in-hospital mortality. These patients may require more aggressive treatment and ICP monitoring to control cerebral edema and intracranial pressure. The APS III score measures the severity of a patient’s acute physiological state, with higher scores reflecting worsening clinical conditions (45). In Groups 2 and 3, APS III scores were higher than in Group 1, and SII levels remained elevated in Group 3. The differences in APS III scores suggest that patients with different SII trajectories not only differ in their inflammatory responses but also show significant variations in the severity of their acute physiological conditions. Patients with sustained high SII levels and higher APS III scores are likely to face a higher risk of in-hospital mortality, highlighting the need for closer monitoring and more aggressive interventions in clinical practice. Patients in Group 1 exhibited a rapid decline in SII after injury and consistently maintained low SII levels. This cohort had the lowest mortality rate, indicating that patients in this category may experience minimal secondary neuroinflammation-related damage post-injury, and their inflammation may resolve more rapidly.

The pathophysiological mechanisms of TBI are heterogeneous and dynamic (21). Neuroinflammation is an important and modifiable cause of secondary injury after TBI, driven by both central and peripheral immune responses (46). After TBI, the body initiates a systemic immune response, leading to significant changes in various immune cells and inflammatory markers (47). There is a strong connection and interaction between the central nervous system and the peripheral immune system (16). Therefore, changes in SII may be driven by the complex interactions between the central nervous system (CNS) and the peripheral immune system. Elevated SII after TBI may indicate an exacerbation of neuroinflammation and systemic inflammatory responses, leading to poor outcomes. The impact of TBI is not limited to the brain; it can cause multi-organ damage and trigger a systemic immune response, including the production of cytokines and chemokines (48). Current potential therapies targeting peripheral immune responses to CNS injury include anti-HMGB1 monoclonal antibodies, which reduce blood–brain barrier disruption, decrease cerebral edema, and limit inflammatory cascades; metformin, which exhibits anti-inflammatory effects by reducing neutrophilia and normalizing the neutrophil-to-lymphocyte ratio; and dexamethasone/hydrocortisone, which reduce the intensity of immune responses through various cells (16). Therefore, aggressive targeted anti-inflammatory treatment, intracranial pressure management, and timely adjustment of surgical strategies are expected to alter SII trajectories, allowing for a rapid decrease in SII levels. In this study, the model demonstrated an AUC of 0.838, indicating good discriminative ability in predicting mortality in patients with moderate to severe TBI. Compared to two recent models using admission SII levels to predict the prognosis of moderate to severe TBI patients (18, 49), our model’s AUC is higher, highlighting the model’s advantages in prognostic prediction and the importance of dynamic SII monitoring. The model’s sensitivity was 75.0%, meaning that in clinical practice, most patients at risk of death could be identified, facilitating early intervention. The specificity was 83.8%, indicating that the model could effectively exclude non-critical cases, reducing unnecessary treatment burdens. Compared to models in the existing literature (75.0%, 83.8% vs. 64.1%, 92.1% and 48.3%, 84.2%), these metrics are at relatively high levels, demonstrating the potential of this model for application in TBI patients. This model will help clinicians identify high-risk patients early after admission and predict the prognosis of patients with TBI. Such identification could prompt surgeons to expedite necessary interventions, implement stratified management, and provide personalized treatments, such as aggressive pharmacological interventions, targeted anti-inflammatory treatment, intracranial pressure management and timely adjustments in surgical strategies. By combining the model’s predictions with other clinical assessment methods, treatment decisions can be further optimized, ultimately improving patient outcomes.

This study has some limitations that are common in large-scale public database studies. First, data missingness and outliers are common owing to routine clinical electronic records, resulting in inconsistent data density when collecting clinical records of SII. Second, variables were extracted using ICD-9 and ICD-10 diagnostic codes, inevitably leading to coding errors or misclassification biases. Third, the sample size limited significant statistical comparisons of mortality rates among groups in sensitivity analyses. Finally, the database did not include neuroimaging data, therefore, this study was unable to extract neuroimaging data for analysis. Although these limitations may somewhat affect the generalizability of the results, we have taken various measures to minimize their impact. For example, we used multiple imputation methods to handle missing data, applied strict inclusion and exclusion criteria as well as sensitivity analyses to enhance the validity and generalizability of the findings, and adjusted for potential confounding factors in the multivariable regression models to reduce bias. Therefore, despite its limitations, the results of this study still hold significant clinical relevance and generalizability to some extent. However, to further validate our conclusions, future research could conduct larger-scale prospective cohort studies, incorporating more comprehensive clinical and imaging data to enhance the validity and generalizability of the findings.

## 5 Conclusion

In summary, this study employed GBTM to identify three dynamic SII trajectories after TBI, offering fresh insights into the interactions between peripheral immune system and secondary brain injury following trauma. Patients in the “sustained high-level” group exhibited the highest risk of all-cause hospital mortality. Early monitoring of the dynamic changes of SII helps identify subtypes with high mortality risk and enhances treatment strategies. SII trajectory modeling contributes to our understanding of the pathology of post-traumatic neuroinflammation and enables more targeted treatment approaches.

## Data Availability

Publicly available datasets were analyzed in this study. This data can be found at: eICU Collaborative Research Database v2.0 https://physionet.org/content/eicu-crd/2.0/.

## Footnotes

^1^ https://github.com/MIT-LCP/eicu-code

## References

[1] Roozenbeek B Maas AIR Menon DK. Changing patterns in the epidemiology of traumatic brain injury. Nat Rev Neurol. (2013) 9:231–6. doi: 10.1038/nrneurol.2013.22, PMID: 23443846

[2] Faul M Xu L Wald MM Coronado VG. Traumatic brain injury in the United States: Emergency department visits, hospitalizations and deaths 2002–2006. Atlanta (GA): Centers for Disease Control and Prevention, National Center for Injury Prevention and Control (2010).

[3] Salehpour F Bazzazi AM Aghazadeh J Hasanloei AV Pasban K Mirzaei F. What do you expect from patients with severe head trauma? Asian J Neurosurg. (2018) 13:660–3. doi: 10.4103/ajns.AJNS_260_16 30283522 PMC6159042

[4] Salehpour F Bazzazi AM Aghazadeh J Abbasivash R Forouhideh Y Mirzaei F. Can serum glucose level in early admission predict outcome in patients with severe head trauma? World Neurosurg. (2016) 87:132–5. doi: 10.1016/j.wneu.2015.11.048, PMID: 26704213

[5] Stevens RD Sutter R. Prognosis in severe brain injury. Crit Care Med. (2013) 41:1104–23. doi: 10.1097/CCM.0b013e318287ee79, PMID: 23528755

[6] Ghaith HS Nawar AA Gabra MD. A literature review of traumatic brain injury biomarkers. Mol Neurobiol. (2022) 59:4141–4158. doi: 10.1007/s12035-022-02822-6, PMID: 35499796 PMC9167167

[7] Corps KN Roth TL McGavern DB. Inflammation and neuroprotection in traumatic brain injury. JAMA Neurol. (2015) 72:355–62. doi: 10.1001/jamaneurol.2014.3558, PMID: 25599342 PMC5001842

[8] Lassarén P Lindblad C Frostell A Carpenter KLH Guilfoyle MR Hutchinson PJA. Systemic inflammation alters the neuroinflammatory response: a prospective clinical trial in traumatic brain injury. J Neuroinflammation. (2021) 18:221. doi: 10.1186/s12974-021-02264-2 34563211 PMC8464153

[9] Russo MV McGavern DB. Inflammatory neuroprotection following traumatic brain injury. Science. (2016) 353:783–5. doi: 10.1126/science.aaf6260, PMID: 27540166 PMC5260471

[10] Hubbard WB Dong J-F Cruz MA Rumbaut RE. Links between thrombosis and inflammation in traumatic brain injury. Thromb Res. (2021) 198:62–71. doi: 10.1016/j.thromres.2020.10.041, PMID: 33290884 PMC7867616

[11] Roth TL Nayak D Atanasijevic T Koretsky AP Latour LL McGavern DB. Transcranial amelioration of inflammation and cell death after brain injury. Nature. (2014) 505:223–8. doi: 10.1038/nature12808, PMID: 24317693 PMC3930079

[12] Martin GE Xia B Kim Y Johnson MD Veile R Friend LA. Platelet function changes in a time-dependent manner following traumatic brain injury in a murine model. Shock. (2018) 50:551–6. doi: 10.1097/SHK.0000000000001056, PMID: 29140832

[13] Alam A Thelin EP Tajsic T Khan DZ Khellaf A Patani R. Cellular infiltration in traumatic brain injury. J Neuroinflammation. (2020) 17:328. doi: 10.1186/s12974-020-02005-x 33143727 PMC7640704

[14] Ge X Zhu L Li M Li W Chen F Li Y. A novel blood inflammatory Indicator for predicting deterioration risk of mild traumatic brain injury. Front Aging Neurosci. (2022) 14:878484. doi: 10.3389/fnagi.2022.878484, PMID: 35557838 PMC9087837

[15] Li W Deng W. Platelet-to-lymphocyte ratio predicts short-term mortality in patients with moderate to severe traumatic brain injury. Sci Rep. (2022) 12:13976. doi: 10.1038/s41598-022-18242-4 35978006 PMC9385644

[16] Duan M Xu Y Li Y Feng H Chen Y. Targeting brain-peripheral immune responses for secondary brain injury after ischemic and hemorrhagic stroke. J Neuroinflammation. (2024) 21:102. doi: 10.1186/s12974-024-03101-y 38637850 PMC11025216

[17] Hu B Yang X-R Xu Y Sun Y-F Sun C Guo W. Systemic immune-inflammation index predicts prognosis of patients after curative resection for hepatocellular carcinoma. Clin Cancer Res. (2014) 20:6212–22. doi: 10.1158/1078-0432.CCR-14-0442, PMID: 25271081

[18] Chen L Xia S Zuo Y Lin Y Qiu X Chen Q. Systemic immune inflammation index and peripheral blood carbon dioxide concentration at admission predict poor prognosis in patients with severe traumatic brain injury. Front Immunol. (2022) 13:1034916. doi: 10.3389/fimmu.2022.1034916, PMID: 36700228 PMC9868584

[19] Xu H Wu W Zhu Q Wang J Ding P Zhuang Z. Systemic immune-inflammation index predicts the prognosis of traumatic brain injury. World Neurosurg. (2024) 183:e22–7. doi: 10.1016/j.wneu.2023.10.081, PMID: 37865196

[20] Sulhan S Lyon KA Shapiro LA. Neuroinflammation and blood-brain barrier disruption following traumatic brain injury: pathophysiology and potential therapeutic targets. J Neurosci Res. (2020) 98:19–28. doi: 10.1002/jnr.24331, PMID: 30259550 PMC6437022

[21] Sabouri E Majdi A Jangjui P Rahigh Aghsan S Naseri Alavi SA. Neutrophil-to-lymphocyte ratio and traumatic brain injury: A review study. World Neurosurg. (2020) 140:142–7. doi: 10.1016/j.wneu.2020.04.185, PMID: 32360917

[22] Nagin DS Odgers CL. Group-based trajectory modeling in clinical research. Annu Rev Clin Psychol. (2010) 6:109–38. doi: 10.1146/annurev.clinpsy.121208.131413, PMID: 20192788

[23] Nagin DS. Group- based modeling of development. Cambridge: Harvard University Press (2005).

[24] Pollard TJ Johnson AEW Raffa JD Celi LA Mark RG Badawi O. The eICU collaborative research database, a freely available multi-center database for critical care research. Sci Data. (2018) 5:180178. doi: 10.1038/sdata.2018.178, PMID: 30204154 PMC6132188

[25] Cai W Xu J Wu X Chen Z Zeng L Song X. Association between triglyceride-glucose index and all-cause mortality in critically ill patients with ischemic stroke: analysis of the MIMIC-IV database. Cardiovasc Diabetol. (2023) 22:138. doi: 10.1186/s12933-023-01864-x 37312120 PMC10262584

[26] Zhao G-J Xu C Ying J-C Lü W-B Hong G-L Li M-F. Association between furosemide administration and outcomes in critically ill patients with acute kidney injury. Crit Care. (2020) 24:75. doi: 10.1186/s13054-020-2798-6 32131879 PMC7057586

[27] Jones BL Nagin DS Roeder K. A SAS procedure based on mixture models for estimating developmental trajectories. Sociol Methods Res. (2001) 29:374–93. doi: 10.1177/0049124101029003005

[28] Kass RE Raftery AE. Bayes factors. J Am Stat Assoc. (1995) 90:773–95. doi: 10.1080/01621459.1995.10476572

[29] Niyonkuru C Wagner AK Ozawa H Amin K Goyal A Fabio A. Group-based trajectory analysis applications for prognostic biomarker model development in severe TBI: a practical example. J Neurotrauma. (2013) 30:938–45. doi: 10.1089/neu.2012.2578, PMID: 23421760

[30] Jha RM Elmer J Zusman BE Desai S Puccio AM Okonkwo DO. Intracranial pressure trajectories: A novel approach to informing severe traumatic brain injury phenotypes. Crit Care Med. (2018) 46:1792–802. doi: 10.1097/CCM.0000000000003361, PMID: 30119071 PMC6185785

[31] Kim S-Y. Determining the number of latent classes in single- and multi-phase growth mixture models. Struct Equ Model. (2014) 21:263–79. doi: 10.1080/10705511.2014.882690 PMC3979564 24729675

[32] Herle M Micali N Abdulkadir M Loos R Bryant-Waugh R Hübel C. Identifying typical trajectories in longitudinal data: modelling strategies and interpretations. Eur J Epidemiol. (2020) 35:205–22. doi: 10.1007/s10654-020-00615-6, PMID: 32140937 PMC7154024

[33] von Leden RE Parker KN Bates AA Noble-Haeusslein LJ Donovan MH. The emerging role of neutrophils as modifiers of recovery after traumatic injury to the developing brain. Exp Neurol. (2019) 317:144–54. doi: 10.1016/j.expneurol.2019.03.004, PMID: 30876905 PMC6544477

[34] McGovern AJ Barreto GE. Mitochondria dysfunction and inflammation in traumatic brain injury: androgens to the battlefront. Androgens: Clin Res Therapeutics. (2021) 2:304–15. doi: 10.1089/andro.2021.0017

[35] Pun PBL Lu J Moochhala S. Involvement of ROS in BBB dysfunction. Free Radic Res. (2009) 43:348–64. doi: 10.1080/10715760902751902, PMID: 19241241

[36] Xu X Kozar R Zhang J Dong J-F. Diverse activities of von Willebrand factor in traumatic brain injury and associated coagulopathy. J Thromb Haemost. (2020) 18:3154–62. doi: 10.1111/jth.15096, PMID: 32931638 PMC7855263

[37] Wang KK Yang Z Zhu T Shi Y Rubenstein R Tyndall JA. An update on diagnostic and prognostic biomarkers for traumatic brain injury. Expert Rev Mol Diagn. (2018) 18:165–80. doi: 10.1080/14737159.2018.1428089 29338452 PMC6359936

[38] Ma F Li L Xu L Wu J Zhang A Liao J. The relationship between systemic inflammation index, systemic immune-inflammatory index, and inflammatory prognostic index and 90-day outcomes in acute ischemic stroke patients treated with intravenous thrombolysis. J Neuroinflammation. (2023) 20:220. doi: 10.1186/s12974-023-02890-y 37777768 PMC10543872

[39] Wang R-H Wen W-X Jiang Z-P Du Z-P Ma Z-H Lu A-L. The clinical value of neutrophil-to-lymphocyte ratio (NLR), systemic immune-inflammation index (SII), platelet-to-lymphocyte ratio (PLR) and systemic inflammation response index (SIRI) for predicting the occurrence and severity of pneumonia in patients with intracerebral hemorrhage. Front Immunol. (2023) 14:1115031. doi: 10.3389/fimmu.2023.1115031 36860868 PMC9969881

[40] Geraghty JR Lung TJ Hirsch Y Katz EA Cheng T Saini NS. Systemic immune-inflammation index predicts delayed cerebral vasospasm after aneurysmal subarachnoid hemorrhage. Neurosurgery. (2021) 89:1071–9. doi: 10.1093/neuros/nyab354, PMID: 34560777 PMC8600162

[41] Stocchetti N Carbonara M Citerio G Ercole A Skrifvars MB Smielewski P. Severe traumatic brain injury: targeted management in the intensive care unit. Lancet Neurol. (2017) 16:452–64. doi: 10.1016/S1474-4422(17)30118-7, PMID: 28504109

[42] Blennow K Brody DL Kochanek PM Levin H McKee A Ribbers GM. Traumatic brain injuries. Nat Rev Dis Primers. (2016) 2:16084. doi: 10.1038/nrdp.2016.84 27853132

[43] Fu S Liu H Wang G Hu X Wang S. Incidence, risk factors, and clinical outcomes of acute brain swelling associated with traumatic acute subdural hematoma: a retrospective study utilizing novel diagnostic criteria. Ther Adv Neurol Disord. (2024) 17:17562864241242944. doi: 10.1177/17562864241242944 38638672 PMC11025420

[44] Jha RM Kochanek PM. A precision medicine approach to cerebral edema and intracranial hypertension after severe traumatic brain injury: quo Vadis? Curr Neurol Neurosci Rep. (2018) 18:105. doi: 10.1007/s11910-018-0912-9 30406315 PMC6589108

[45] Knaus WA Wagner DP Draper EA Zimmerman JE Bergner M Bastos PG. The APACHE III prognostic system. risk prediction of hospital mortality for critically ill hospitalized adults. Chest. (1991) 100:1619–36. doi: 10.1378/chest.100.6.1619, PMID: 1959406

[46] Duchniewicz M Lee JYW Menon DK Needham EJ. Candidate genetic and molecular drivers of dysregulated adaptive immune responses after traumatic brain injury. J Neurotrauma. (2024) 41:3–12. doi: 10.1089/neu.2023.0187, PMID: 37376743

[47] Rhind SG Crnko NT Baker AJ Morrison LJ Shek PN Scarpelini S. Prehospital resuscitation with hypertonic saline-dextran modulates inflammatory, coagulation and endothelial activation marker profiles in severe traumatic brain injured patients. J Neuroinflammation. (2010) 7:5. doi: 10.1186/1742-2094-7-5, PMID: 20082712 PMC2819256

[48] Das M Mohapatra S Mohapatra SS. New perspectives on central and peripheral immune responses to acute traumatic brain injury. J Neuroinflammation. (2012) 9:1–12. doi: 10.1186/1742-2094-9-236, PMID: 23061919 PMC3526406

[49] Arslan K Sahin AS. Prognostic value of systemic immune-inflammation index, neutrophil-lymphocyte ratio, and thrombocyte-lymphocyte ratio in critically ill patients with moderate to severe traumatic brain injury. Medicine (Baltimore). (2024) 103:e39007. doi: 10.1097/MD.0000000000039007, PMID: 39029062 PMC11398738

