# Supplementary material for: Trajectories of systemic immune inflammation index and mortality risk in patients with moderate-to-severe traumatic brain injury: a retrospective cohort study
Source: Front Neurol. 2025 Feb 12;15:1439318. doi: 10.3389/fneur.2024.1439318 (PMC11860105; doi:10.3389/fneur.2024.1439318)
Supplement: Supplementary file 1 [file Data_Sheet_1.docx]

Supplementary Material

# Supplementary Figures and Tables

## Supplementary Figures Supplementary Figure 1. The distinct trajectory groups from one group to six groups in the selection process of GBTM.

##
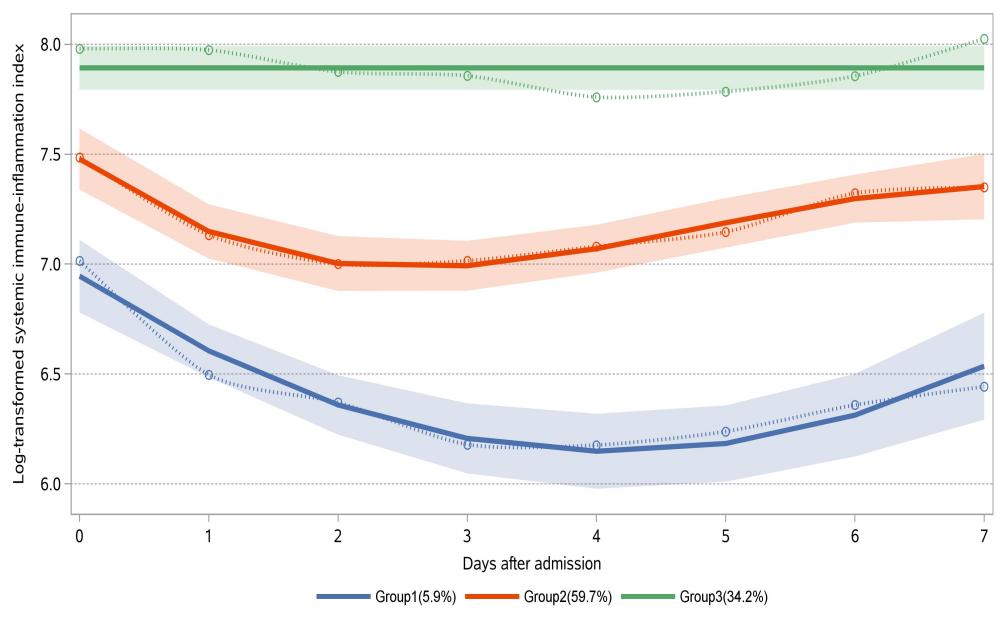


## Supplementary Figure 2. Sensitivity analysis: Excluding patients with GCS>8, the SII trajectories within the first 7 days post-admission following trauma were defined using group-based trajectory modeling.

## 1.2 Supplementary Tables

Supplementary Table 1. Missing number (%) for included variables in the dataset.

| **Variables** | **Missing, N (%)** |
| --- | --- |
| Ethnicity | 0.32% |
| BMI | 0.64% |
| Min creatinine | 2.56% |
| Max creatinine | 2.56% |
| Min glucose | 0.64% |
| Max glucose | 0.64% |
| Min Hb | 2.56% |
| Max Hb | 2.56% |
| Min INR | 9.62% |
| Max INR | 9.62% |
| Min platelet | 2.56% |
| Max platelet | 2.56% |
| Min potassium | 1.60% |
| Max potassium | 1.60% |
| Min sodium | 2.24% |
| Max sodium | 2.24% |
| Min WBC | 2.56% |
| Max WBC | 2.56% |
| Min NEU | 5.45% |
| Max NEU | 5.45% |
| Min LYM | 5.13% |
| Max LYM | 5.13% |
| Min MON | 5.45% |
| Max MON | 5.45% |
| Min SBP | 1.28% |
| Max SBP | 1.28% |
| Min DBP | 1.28% |
| Max DBP | 1.28% |
| APSIII | 3.53% |

BMI, body mass index; Hb, hemoglobin; INR, International Normalized Ratio;

WBC, white blood cells; NEU, neutrophile; LYM, lymphocyte; MON, monocyte;

SBP, systolic blood pressure; DBP, diastolic blood pressure;

APSIII, acute physiology score III

**Supplementary Table 2.** The selection process of GBTM.

| Group | Avepp(%) | OCC | BIC | AIC | LL |
| --- | --- | --- | --- | --- | --- |
| 1Group (2 ) | 100.00 | . | -1978.76 | -1967.82 | -1963.82 |
| 2Group (2 2 ) | 92.25;92.21 | 15.3;9.2 | -1784.19 | -1762.31 | -1754.31 |
| 3Group (2 3 0 ) | 87.59;88.61;91.51 | 28.5;5.7;36.8 | -1711.14 | -1681.05 | -1670.05 |
| 4Group (1 3 3 0 ) | 93.75;87.10;85.99;87.43 | 397.4;15.7;6.6;31.8 | -1679.93 | -1638.89 | -1623.89 |
| 5Group (1 3 1 3 1 ) | 96.79;86.93;86.81;84.35;88.14 | 766.2;13.7;72.2;7.0;57.5 | -1658.61 | -1606.64 | -1587.64 |
| 6Group (0 2 3 1 1 1 ) | 97.57;87.27;82.00;85.26;84.45;89.97 | 1489.2;21.1;6.1;62.9;25.8;208.5 | -1648.25 | -1593.54 | -1573.54 |

Avepp, Average posterior probability; OCC, Odds of Correct Classification; BIC, Bayesian Information Criterion; AIC, Akaike information criterion; LL, Log-likelihood

**Supplementary Table 3.** Comparison of characteristics in survivors and non-survivors.

|  | ALL   N=312 | NO   N=260 | YES   N=52 | p |
| --- | --- | --- | --- | --- |
|  |  |  |  |  |
| Demographics |  |  |  |  |
| Age(years,median [IQR]) | 54.0 [32.8;70.0] | 53.0 [33.8;70.0] | 56.5 [32.0;66.5] | 0.928 |
| Gender,n (%) |  |  |  | 0.707 |
| Female | 104 (33.3%) | 85 (32.7%) | 19 (36.5%) |  |
| Male | 208 (66.7%) | 175 (67.3%) | 33 (63.5%) |  |
| Ethnicity,n (%) |  |  |  | 0.75 |
| African American | 21 (6.73%) | 18 (6.92%) | 3 (5.77%) |  |
| Asian | 5 (1.60%) | 3 (1.15%) | 2 (3.85%) |  |
| Caucasian | 241 (77.2%) | 200 (76.9%) | 41 (78.8%) |  |
| Hispanic | 21 (6.73%) | 18 (6.92%) | 3 (5.77%) |  |
| Native American | 2 (0.64%) | 2 (0.77%) | 0 (0.00%) |  |
| Other/Unknown | 22 (7.05%) | 19 (7.31%) | 3 (5.77%) |  |
| BMI(kg/m2,median[IQR]) | 26.4 [22.9;29.9] | 25.9 [22.8;29.7] | 27.0 [23.3;32.0] | 0.265 |
| CCI,median(IQR) | 2.00 [0.00;4.00] | 2.00 [0.00;4.00] | 3.00 [1.00;4.00] | 0.164 |
| Laboratory variates,median(IQR) |  |  |  |  |
| Min creatinine (mg/dl) | 0.78 [0.63;0.95] | 0.77 [0.63;0.92] | 0.82 [0.63;1.01] | 0.302 |
| Max creatinine (mg/dl) | 0.92 [0.78;1.14] | 0.90 [0.77;1.10] | 1.09 [0.83;1.43] | **0.003** |
| Min glucose (mmol/L) | 108 [93.0;127] | 107 [94.8;127] | 111 [88.0;133] | 0.95 |
| Max glucose (mmol/L) | 160 [135;202] | 155 [133;195] | 188 [157;244] | **<0.001** |
| Min Hb (g/dl) | 11.4 [9.30;12.7] | 11.5 [9.57;12.9] | 10.7 [8.47;12.1] | **0.02** |
| Max Hb (g/dl) | 13.3 [11.8;14.6] | 13.2 [11.9;14.7] | 13.3 [11.7;14.2] | 0.404 |
| Min INR (ratio) | 1.10 [1.00;1.20] | 1.10 [1.00;1.20] | 1.20 [1.01;1.30] | **0.003** |
| Max INR (ratio) | 1.17 [1.09;1.30] | 1.13 [1.09;1.30] | 1.30 [1.10;1.61] | **0.002** |
| Min platelet (109/L) | 174 [134;223] | 176 [141;228] | 166 [107;201] | **0.032** |
| Max platelet (109/L) | 223 [178;280] | 223 [180;278] | 224 [163;282] | 0.829 |
| Min potassium (mmol/L) | 3.50 [3.20;3.80] | 3.50 [3.27;3.80] | 3.50 [3.10;3.80] | 0.347 |
| Max potassium (mmol/L) | 4.10 [3.90;4.60] | 4.10 [3.88;4.50] | 4.25 [3.98;4.70] | 0.1 |
| Min sodium (mmol/L) | 138 [136;140] | 138 [136;140] | 138 [135;141] | 0.643 |
| Max sodium (mmol/L) | 142 [139;145] | 142 [139;144] | 145 [140;149] | **<0.001** |
| Min WBC (109/L) | 10.5 [8.13;13.7] | 10.2 [8.07;13.4] | 11.7 [8.75;15.7] | 0.06 |
| Max WBC (109/L) | 15.2 [10.8;18.9] | 15.0 [10.4;18.2] | 17.8 [11.8;23.8] | **0.016** |
| Min NEU (109/L) | 8.02 [5.65;10.9] | 7.71 [5.62;10.5] | 9.41 [7.23;12.4] | **0.026** |
| Max NEU (109/L) | 11.4 [8.13;15.3] | 11.2 [8.01;14.8] | 13.1 [8.59;18.3] | 0.05 |
| Min LYM (109/L) | 1.02 [0.68;1.46] | 1.02 [0.69;1.49] | 0.98 [0.58;1.29] | 0.22 |
| Max LYM (109/L) | 1.73 [1.13;2.97] | 1.73 [1.12;2.80] | 1.75 [1.22;3.94] | 0.337 |
| Min MON (109/L) | 0.72 [0.53;0.97] | 0.72 [0.53;0.95] | 0.78 [0.58;1.10] | 0.111 |
| Max MON (109/L) | 1.00 [0.74;1.40] | 0.99 [0.75;1.34] | 1.11 [0.69;1.67] | 0.275 |
| Vital Signs,median(IQR) |  |  |  |  |
| Min heartrate (times/minute) | 67.0 [58.0;77.0] | 67.0 [58.0;76.2] | 64.5 [56.8;78.5] | 0.912 |
| Max heartrate (times/minute) | 110 [96.8;128] | 110 [95.0;125] | 124 [107;143] | **0.001** |
| Min SBP (mmHg) | 97.7 (21.7) | 99.8 (20.8) | 86.8 (23.1) | **<0.001** |
| Max SBP (mmHg) | 161 [147;180] | 160 [147;177] | 170 [148;185] | 0.107 |
| Min DBP (mmHg) | 50.5 (13.2) | 51.6 (12.6) | 45.1 (14.8) | **0.004** |
| Max DBP (mmHg) | 97.5 [84.0;113] | 97.0 [85.0;112] | 100 [82.0;114] | 0.944 |
| Min temperature (℃) | 36.3 [35.7;36.7] | 36.3 [35.8;36.7] | 35.8 [34.8;36.4] | **<0.001** |
| Max temperature (℃) | 38.0 (0.72) | 38.0 (0.69) | 38.1 (0.84) | 0.645 |
| Type of TBI,n (%) |  |  |  |  |
| Skull_Fracture |  |  |  | 0.703 |
| no | 299 (95.8%) | 250 (96.2%) | 49 (94.2%) |  |
| yes | 13 (4.17%) | 10 (3.85%) | 3 (5.77%) |  |
| EDH |  |  |  | >0.999 |
| no | 295 (94.6%) | 246 (94.6%) | 49 (94.2%) |  |
| yes | 17 (5.45%) | 14 (5.38%) | 3 (5.77%) |  |
| SAH |  |  |  | 0.08 |
| no | 215 (68.9%) | 185 (71.2%) | 30 (57.7%) |  |
| yes | 97 (31.1%) | 75 (28.8%) | 22 (42.3%) |  |
| SDH |  |  |  | 0.918 |
| no | 187 (59.9%) | 155 (59.6%) | 32 (61.5%) |  |
| yes | 125 (40.1%) | 105 (40.4%) | 20 (38.5%) |  |
| ICH |  |  |  | 0.761 |
| no | 292 (93.6%) | 244 (93.8%) | 48 (92.3%) |  |
| yes | 20 (6.41%) | 16 (6.15%) | 4 (7.69%) |  |
| Contusion OR Laceration |  |  |  | 0.095 |
| no | 230 (73.7%) | 197 (75.8%) | 33 (63.5%) |  |
| yes | 82 (26.3%) | 63 (24.2%) | 19 (36.5%) |  |
| Scoring Systems,median(IQR) |  |  |  |  |
| Min GCS | 6.00 [3.00;8.00] | 6.00 [3.00;8.00] | 3.00 [3.00;6.00] | **0.002** |
| SOFA | 4.00 [3.00;6.00] | 4.00 [3.00;5.25] | 6.00 [4.00;8.25] | **<0.001** |
| APSIII | 53.0 [37.0;74.0] | 49.0 [33.8;69.2] | 78.0 [52.0;100] | **<0.001** |
| Clinical Treatments,n (%) |  |  |  |  |
| Ventriculostomy |  |  |  | **0.022** |
| no | 273 (87.5%) | 233 (89.6%) | 40 (76.9%) |  |
| yes | 39 (12.5%) | 27 (10.4%) | 12 (23.1%) |  |
| Craniotomy |  |  |  | >0.999 |
| no | 287 (92.0%) | 239 (91.9%) | 48 (92.3%) |  |
| yes | 25 (8.01%) | 21 (8.08%) | 4 (7.69%) |  |
| Csf drainage |  |  |  | 0.154 |
| no | 286 (91.7%) | 241 (92.7%) | 45 (86.5%) |  |
| yes | 26 (8.33%) | 19 (7.31%) | 7 (13.5%) |  |
| Craniectomy |  |  |  | >0.999 |
| no | 308 (98.7%) | 257 (98.8%) | 51 (98.1%) |  |
| yes | 4 (1.28%) | 3 (1.15%) | 1 (1.92%) |  |
| Surgery |  |  |  | 0.069 |
| no | 248 (79.5%) | 212 (81.5%) | 36 (69.2%) |  |
| yes | 64 (20.5%) | 48 (18.5%) | 16 (30.8%) |  |
| Hypertonic saline |  |  |  | 0.167 |
| no | 268 (85.9%) | 227 (87.3%) | 41 (78.8%) |  |
| yes | 44 (14.1%) | 33 (12.7%) | 11 (21.2%) |  |
| Mannitol |  |  |  | **<0.001** |
| no | 284 (91.0%) | 244 (93.8%) | 40 (76.9%) |  |
| yes | 28 (8.97%) | 16 (6.15%) | 12 (23.1%) |  |
| Hospital Outcomes |  |  |  |  |
| hosp_los_hours | 249 [148;420] | 276 [162;471] | 158 [93.5;244] | **<0.001** |
| SII Characteristics,median(IQR) |  |  |  |  |
| SII_avg | 1557 [1014;2236] | 1474 [959;2079] | 1938 [1419;2593] | **<0.001** |
| SII_min | 782 [546;1123] | 736 [506;1072] | 1032 [688;1519] | **0.002** |
| SII_max | 2622 [1666;4216] | 2438 [1578;4167] | 3151 [2333;4487] | **0.017** |

BMI, body mass index; CCI, Charlson comorbidity index; Hb, hemoglobin; INR, International Normalized Ratio; WBC, white blood cells; NEU, neutrophile; LYM, lymphocyte; MON, monocyte; SBP, systolic blood pressure; DBP, diastolic blood pressure; TBI, traumatic brain injury; EDH, epidural hematoma; SDH, subdural hematoma; SAH, subarachnoid hemorrhage; ICH, intracerebral hemorrhage; GCS, Glasgow Coma Score; SOFA, sequential organ failure assessment; APSIII, acute physiology score III; CSF, cerebrospinal fluid; LOS, length of hospital stay; SII, systemic immune inflammation index

Boldface type indicates P < 0.05.

Supplementary Table 4. Sensitivity analysis: Association between trajectory groups and all-cause hospital mortality.

| Trajectories | Multivariate Analysis |  |
| --- | --- | --- |
|  | OR (95% CI) | p |
| Group 1 | Reference |  |
| Group 2 | 1.46(0.27,13.70) | 0.696 |
| Group 3 | 3.10(0.56,29.33) | 0.245 |

Adjusted by APSIII, Mannitol, Min SBP, Max creatinine, Age, Min GCS, Surgery. OR, odds ratio; CI, confidence interval. Boldface type indicates P < 0.05.
